# Supplementary material for: Swimming motility of a gut bacterial symbiont promotes resistance to intestinal expulsion and enhances inflammation
Source: PLoS Biol. 2020 Mar 20;18(3):e3000661. doi: 10.1371/journal.pbio.3000661 (PMC7112236; doi:10.1371/journal.pbio.3000661)
Supplement: S1 Table — (PDF) [file pbio.3000661.s015.pdf]

S1 Table. Bacteria used and created in this study.

Strain	Description/ Relevant Details	Source
**Wild-type Bacteria**		
Vibrio ZWU0020	Non-toxigenic strain of *Vibrio cholerae* isolated from the zebrafish gut. IMG genome ID: 2703719078	[1]
**Recombinant Bacteria**		
Vibrio ∆mot	*Vibrio* ZWU0020 with unmarked, in-frame deletion of *pomAB* (non-motile)	[2]
Vibrio ∆che	*Vibrio* ZWU0020 with unmarked, in-frame deletion of *cheA2* (non-chemotactic)	This study
Aeromonas ZOR0001 *attTn7*::*dTomato*	Strain of *Aeromonas veronii* isolated from the zebrafish gut. Constitutively expresses dTomato; $Gent^R$	[2]
Vibrio ZWU0020 *attTn7*::*dTomato*	constitutively expresses dTomato; $Gent^R$	[2]
Vibrio ZWU0020 *attTn7*::*sfGFP*	constitutively expresses sfGFP; $Gent^R$	[2]
Vibrio ∆mot *attTn7*::*dTomato*	constitutively expresses dTomato; $Gent^R$	This study
Vibrio ∆che *attTn7*::*dTomato*	constitutively expresses dTomato; $Gent^R$	This study
$\textit{Vibrio}^{motLOF}$	*Vibrio* carrying the motility loss-of-function switch within the chromosome at the *attTn7* site; constitutively expresses dTomato; $Gent^R$	This study
Vibrio Δmot^{GOF}	∆mot carrying the motility gain-of-function switch within the chromosome at the *attTn7* site; constitutively expresses dTomato; $Gent^R$	This study
Vibrio Δche^{GOF}	∆che carrying the chemotaxis gain-of-function switch within the chromosome at the *attTn7* site; constitutively expresses dTomato; $Gent^R$	This study
**Other bacteria used for molecular biology and switch optimization**		
E. coli SM10	Donor strain used for conjugation	[3]
DH5α	*E. coli* cloning strain	NEB
E. coli MG1655	Used for switch prototyping and optimization	[4]
E. coli HS	Used for switch prototyping and optimization	[5]
Enterobacter ZOR0014	Source of *tetR* gene	[1]

REFERENCES

1. Stephens WZ, Burns AR, Stagaman K, Wong S, Rawls JF, Guillemin K, et al. The composition of the zebrafish intestinal microbial community varies across development. ISME J. 2016;10: 644–654. doi:10.1038/ismej.2015.140
2. Wiles TJ, Wall ES, Schlomann BH, Hay EA, Parthasarathy R, Guillemin K. Modernized tools for streamlined genetic manipulation and comparative study of wild and diverse proteobacterial lineages. MBio. American Society for Microbiology; 2018;9. doi:10.1128/mBio.01877-18
3. Simon R, Priefer U, Pühler A. A Broad Host Range Mobilization System for In Vivo Genetic Engineering: Transposon Mutagenesis in Gram Negative Bacteria. Bio/Technology. Nature Publishing Group; 1983;1: 784–791. doi:10.1038/nbt1183-784
4. Blattner FR, Plunkett G, Bloch CA, Perna NT, Burland V, Riley M, et al. The complete genome sequence of Escherichia coli K-12. Science. 1997;277: 1453–62. Available: http://www.ncbi.nlm.nih.gov/pubmed/9278503
5. Rasko DA, Rosovitz MJ, Myers GSA, Mongodin EF, Fricke WF, Gajer P, et al. The Pangenome Structure of Escherichia coli: Comparative Genomic Analysis of E. coli Commensal and Pathogenic Isolates. J Bacteriol. 2008;190: 6881–6893. doi:10.1128/JB.00619-08
