# Supplementary material for: Swimming motility of a gut bacterial symbiont promotes resistance to intestinal expulsion and enhances inflammation
Source: PLoS Biol. 2020 Mar 20;18(3):e3000661. doi: 10.1371/journal.pbio.3000661 (PMC7112236; doi:10.1371/journal.pbio.3000661)
Supplement: S2 Table — (PDF) [file pbio.3000661.s016.pdf]

S2 Table. Plasmids used and created in this study.

Plasmid Name	Description/ Relevant Details	Source
**Plasmids used for allelic exchange**		
pAX1	allelic exchange vector with GFP merodiploid tracker and temperature-sensitive replicon *ori*$_{101}$/*repA101*^{ts}; Amp^R, Gent^R, Clm^R, 30°C; Addgene plasmid #117397	[1]
pAX1-ZWU0020-cheA2	pAX1-based plasmid with *cheA2* knockout cassette; Amp^R, Gent^R, Clm^R, 30°C; Wiles plasmid #pTW383	This study
**Customizable plasmid-based genetic switch scaffolds**		
pXS-GOF-switch	gain-of-function switch scaffold constructed in a pXS-based plasmid; Amp^R; Wiles plasmid #pTW265	This study
pXS-LOF-switch	loss-of-function switch scaffold constructed in a pXS-based plasmid; Amp^R; Wiles plasmid #pTW308	This study
**Plasmids used for Tn*7*-based chromosomal insertions**		
pTn7xTS	Tn7 tagging vector with temperature-sensitive replicon *ori*$_{101}$/*repA101*^{ts}; Amp^R, Gent^R, 30°C; Addgene plasmid #117389	[1]
pTNS2	Tn*7* helper plasmid carrying transposase genes; Amp^R; Addgene plasmid #64968	[2]
pTn7xTS-dTomato	pTn7xTS carrying P$_{tac}$-*dTomato*; Amp^R, Gent^R, 30°C; Addgene plasmid #117391	[1]
pTn7xTS-GOF-switch	pTn7xTS carrying the gain-of-function switch scaffold; Amp^R, Gent^R, 30°C; Wiles plasmid #pTW285	This study
pTn7xTS-LOF-switch	pTn7xTS carrying the loss-of-function switch scaffold; Amp^R, Gent^R, 30°C; Wiles plasmid #pTW317	This study
pTn7xTS-mot-LOF-switch	pTn7xTS-LOF-switch with *pomA* sgRNA; Amp^R, Gent^R, 30°C; Wiles plasmid #pTW340	This study
pTn7xTS-mot-GOF-switch	pTn7xTS-GOF-switch with *pomAB*; Amp^R, Gent^R, 30°C; Wiles plasmid #pTW324	This study
pTn7xTS-che-GOF-switch	pTn7xTS-GOF-switch with *cheA2*; Amp^R, Gent^R, 30°C; Wiles plasmid #pTW282	This study
**Backbones and sources of genetic parts**		
pXS-dTomato	pXS-based modular *dTomato* expression scaffold; Amp^R; Addgene plasmid #117387	[1]
pdCAS9-bacteria	Source vector for *dcas9* gene; Clm^R; Addgene plasmid #44249	[3]
pTW168	Source vector for *sfGFP* gene; Amp^R, Gent^R; Wiles plasmid #pTW168	[4]

Amp^R, encodes ampicillin resistance; Gent^R, encodes gentamicin resistance; Clm^R, encodes chloramphenicol resistance; 30°C, permissive growth temperature

REFERENCES

1. Wiles TJ, Wall ES, Schlomann BH, Hay EA, Parthasarathy R, Guillemin K. Modernized tools for streamlined genetic manipulation and comparative study of wild and diverse proteobacterial lineages. MBio. American Society for Microbiology; 2018;9. doi:10.1128/mBio.01877-18
2. Choi K-H, Gaynor JB, White KG, Lopez C, Bosio CM, Karkhoff-Schweizer RR, et al. A Tn7-based broad-range bacterial cloning and expression system. Nat Methods. 2005;2: 443–448. doi:10.1038/nmeth765
3. Qi LS, Larson MH, Gilbert LA, Doudna JA, Weissman JS, Arkin AP, et al. Repurposing CRISPR as an RNA-γuided platform for sequence-specific control of gene expression. Cell. 2013;152: 1173–1183. doi:10.1016/j.cell.2013.02.022
4. Schlomann BH, Wiles TJ, Wall ES, Guillemin K, Parthasarathy R. Sublethal antibiotics collapse gut bacterial populations by enhancing aggregation and expulsion. Proc Natl Acad Sci. National Academy of Sciences; 2019; 201907567. doi:10.1073/pnas.1907567116
