# Supplementary material for: Swimming motility of a gut bacterial symbiont promotes resistance to intestinal expulsion and enhances inflammation
Source: PLoS Biol. 2020 Mar 20;18(3):e3000661. doi: 10.1371/journal.pbio.3000661 (PMC7112236; doi:10.1371/journal.pbio.3000661)
Supplement: S3 Table — (PDF) [file pbio.3000661.s017.pdf]

S3 Table. Primer and oligo DNA sequences.

Name	Sequence (5'–3')
**Primers**	
WP11	cacgcccctctttaatacga
WP12	agggtaccgatgttgaccag
WP92	gcggccgcggattttaggggtaacgtatg
WP93	gcggccgcccttatcgttgtgcagctaatca
WP118	tgagagctcgaattgggggatccactagtaa
WP138	tgacctaggtccctatcagtgatagagattgacatccctatcagtgatagagatactgagcacaaccg<br>gtagcatgcacccgggttaactttatcaaggagactaaatcatgagca
WP139	tgagtcgaaaaaaaaagcccgctcattaggcgggctttttttattaagacccactttcacatttaag
WP146	tgagcggccgcactcaagaacaataatndrrdnattacatcatgtctagattagataaaagtaaagtg
WP165	tctattcacacgcgcaaaag
WP166	cttatcgttgtgcagctaatcacatacgttacccctaaaatcca
WP167	tggattttaggggtaacgtatgtgattagctgcacaacgataag
WP168	gagcccgacttctaccaaca
WP169	gcgcaaaaagaacaaaaagc
WP170	tgaaagctttagcctgaggagtgcttcgtg
WP171	tgaggtaccttattgttgtccaccgcctagtg
CheA2.ZW20.KOconfirm.REV	gcgattcggagttgatgatt
**gBlocks**	
RiboJ	cacgtgatgaaagcttaatgggtaccatgaagctgtcaccggatgtgctttccggtctgatgagtccg<br>tgaggacgaaacagcctctacaaataattttgtttaatgtagctagctgaagcatgc
CP25-*lacZ* sgRNA	tagcctaggagggcccagcgatcgcattaattaaactttggcagtttattcttgacatgtagtgaggg<br>ggctggtataatcacatagttgggaagggcgatcggtgcgttttagagctagaaatagcaagttaaaa<br>taaggctagtccgttatcaacttgaaaaagtggcaccgagtcggtgctttttttaggcgcgccaatcga<br>tagccggcaggccggcctag
CP25-*pomA* sgRNA	tgctgcttaattaaactttggcagtttattcttgacatgtagtgagggggctggtataatcacataga<br>aatccacgaagcactcctcgttttagagctagaaatagcaagttaaaataaggctagtccgttatcaa<br>cttgaaaaagtggcaccgagtcggtgctttttttagccggctgctgc
