# Supplementary material for: Swimming motility of a gut bacterial symbiont promotes resistance to intestinal expulsion and enhances inflammation
Source: PLoS Biol. 2020 Mar 20;18(3):e3000661. doi: 10.1371/journal.pbio.3000661 (PMC7112236; doi:10.1371/journal.pbio.3000661)
Supplement: S1 Raw images — (PDF) [file pbio.3000661.s019.pdf]

S1A Fig

Δmot construction

ethidium bromide stained agarose gel illuminated
on a UV box and imaged using an iPhone 6S

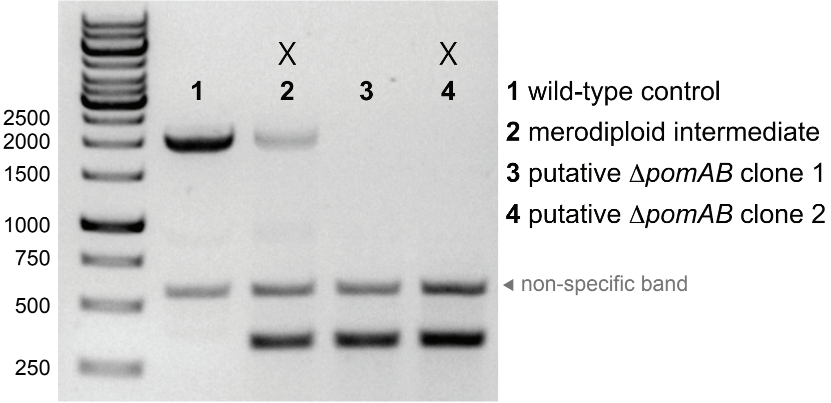

S1A Fig

Δche construction

ethidium bromide stained agarose gel illuminated
on a UV box and imaged using an iPhone 6S

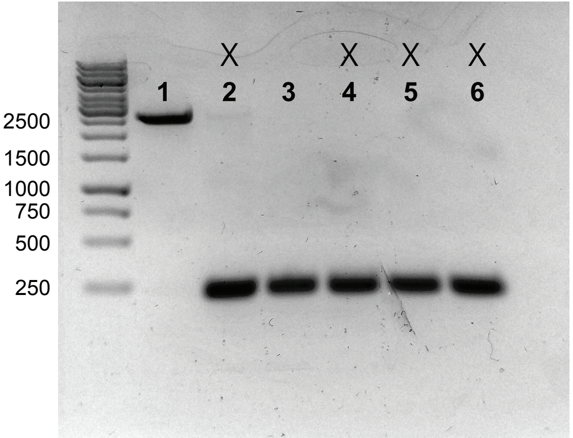

1 wild-type control

2 merodiploid intermediate

3 putative Δ*cheA2* clone 1

4 putative Δ*cheA2* clone 2

5 putative Δ*cheA2* clone 3

6 putative Δ*cheA2* clone 4
