# Supplementary material for: Swimming motility of a gut bacterial symbiont promotes resistance to intestinal expulsion and enhances inflammation
Source: PLoS Biol. 2020 Mar 20;18(3):e3000661. doi: 10.1371/journal.pbio.3000661 (PMC7112236; doi:10.1371/journal.pbio.3000661)
Supplement: Attachment — Submitted filename: renamed_ed70b.pdf [file pbio.3000661.s020.pdf]

UNIVERSITY OF OREGON

February 3, 2020

Dear Dr. Richardson,

Thank you for conducting the review of our manuscript "*Swimming motility of a gut bacterial symbiont promotes resistance to intestinal expulsion and enhances inflammation*" (PBIOLOGY-D-19-03518R1). A revised version of our manuscript has been uploaded to the *PLOS Biology* website and our responses to the reviewers' comments are provided below. We have also made several formatting changes in accordance with the "PLOS formatting requests" document and uploaded a "striking image" for your consideration.

We appreciate the reviewers' thoughtful and constructive comments and thank them for encouraging us to revisit some key issues. We believe we have addressed the reviewers' concerns and had the opportunity to improve the clarity and impact of our study.

Sincerely,

Karen Guillemin, PhD
Philip H. Knight Chair | Professor of Biology | CIFAR Senior Fellow, Humans and the Microbiome Program
University of Oregon | Eugene, OR 97403-1229 | (541) 346-5360 | kguillem@uoregon.edu

An equal-opportunity, affirmative-action institution committed to cultural diversity and compliance with the Americans with Disabilities Act

Reviewer 1 comments

Will Ludington, signed review

1) *line 91 "spacing-filling" --> "space-filling"*

RESPONSE: The text has been revised (p4, line 99).

2) *line 150 It is puzzling that the motility defect also slows entry of cells into the intestine. Is there a hypothesis for why? Maybe this could be addressed in the discussion?*

RESPONSE: Previous work from our group has indicated that bacterial motility traits facilitate dispersal throughout the aqueous environment that zebrafish live in and the transmission of bacterial cells between hosts. We have revised the text within the results to describe this connection and cited the appropriate references:

(p6, lines 145–149)

"Both mutants were slow to access the zebrafish intestine and reached maximal abundances at 24 hpi that were 10–100-fold lower than wild type (Fig 1A). This observation suggests that each mutant has a reduced immigration rate, which is in line with previous work indicating that bacterial motility traits may facilitate dispersal and initial colonization of the zebrafish intestine [37,38]."

3) *line 190 Good summary. Please give a little more indication of the hypothesis for how chemotaxis affects in vivo function of Vibrio. Is it simply that Δche cells can swim but don't?*

RESPONSE: We currently do not know the mechanism by which the chemotaxis mutant becomes aggregated within the zebrafish intestine. There are several possibilities. First, it could be that localization to the anterior, and not simply motility, is necessary to resist intestinal transport, and chemotaxis enables wild-type *Vibrio* to access that niche. Alternatively, chemotaxis may simply enable *Vibrio* to make adjustments to its swimming trajectory, which allow it to avoid entrapment within the matrix of intestinal mucus. Finally, it is possible that chemotaxis mutants experience altered metabolism or induce biofilm-forming behaviors in vivo as a consequence of their inability to effectively control their spatial distribution. To better acknowledge these scenarios and highlight the possibilities for how chemotaxis may be affecting *Vibrio* in vivo, we have made the following revisions within the results section and included the appropriate citations:

(p22, lines 600–618)

"The aggregation of Δmot and Δche within the intestine was unexpected, especially for Δche, which displays vigorous swimming in vitro. It is possible that both mutants experience shifts in metabolism or induce biofilm behaviors in vivo as a consequence of their inability to effectively control their spatial distribution. It is also possible that the aggregation of Δmot and Δche stems from a defect in flagellar assembly or signaling, which could be disentangled using mutants lacking flagella altogether. Alternatively, Δmot and Δche cells may become entrapped within intestinal mucus and grow locally to produce small clonal aggregates that are subsequently consolidated into larger aggregates by intestinal mechanics before being collectively expelled. Corroborating this idea, it was shown in an infant mouse model that the attenuated colonization of a human-derived isolate of *V. cholerae* lacking motility can largely be reversed in animals that are pre-treated with a mucolytic agent that disrupts mucus architecture [56]. Furthermore, it is possible that the swimming activity of Δche may actually facilitate mucus entrapment. It has been shown that non-chemotactic, straight-swimming bacterial cells that are unable to periodically redirect their swimming trajectory can become jammed within a porous medium (such as in a soft agar matrix commonly used to study chemotaxis in vitro) [57]. We propose that a similar mechanism could lead to entrapment and aggregation of Δche cells within intestinal mucus, which wild-type *Vibrio* cells avoid because they are able to actively escape from mucus through regular changes in swimming direction mediated by chemotactic signaling."

4) *line 299-300: What is the growth rate as a function of the bacterial abundance? Growth rates could increase in populations that are turning over faster.*

RESPONSE: We have previously shown that population growth in the zebrafish gut is well-described by a conventional logistic growth function (Jemielita et al, 2014; PMID: 25516613). In our study we report growth rates in low density populations (i.e., populations that begin at least 1 decade below median carrying capacity) in which we expect density-dependent regulation to be low. To clarify this point, we have replaced "growth rate" in the text with "exponential growth rate" on lines 112, 298–300, and 567. The point that "growth rates could increase in populations that are turning over faster" is a good one, though with clarification of "exponential growth rate" in the text, we feel it is not necessary to elaborate further.

5) *line 317 Similar comment to line 190: please hypothesize why chemotaxis blocks aggregation -- or is it just a loss of motility?*

RESPONSE: As outlined in our response to comment #3, we revised the text on p22, lines 600–618 to communicate the possibility that chemotaxis may enable *Vibrio* to localize to an intestinal niche that promotes motility or, alternatively, enable it to avoid physical entrapment within mucus.

6) *Fig 5C,D. Why is there such a high aggregation at the anterior end? Are those adherent? I would expect more even distribution for motile cells such as in Fig. 6A wt cells.*

RESPONSE: The mechanism that produces the pattern of spatial redistribution observed in the time series shown in Fig 5C,D still needs to be fully resolved. One motivation for the induction experiment shown in this figure is that it allowed us to explore the consequences of changing bacterial behavior in vivo *after* the cells had already colonized and assembled their population within the intestine. We did in fact anticipate the possibility that novel structures or behaviors could manifest that were not observed in either pure wild-type or mutant populations. However, many of the features shown in Fig 5D—which is the in vivo complementation of Δche using the chemotaxis gain-of-function switch—can be explained after considering other data presented throughout our study. First, we noted previously in Fig 2C 'Δche' and in the text on p9, lines 244–245 that the chemotaxis mutant appears to form an adherent (or closely associated) layer of cells along the anterior wall of the foregut, which can also be seen in S1 Mov (starting at 00:18) and S2 Mov (starting at 00:38). A similar population is in fact discernible in Fig 5D '3h', which morphs and expands in Fig 5D '4.2h'. In Fig 5D panels labeled '7.2h' and '8.7h', the anterior-localized population grows in size, presumably due, in part, to cells relocating from the midgut region. During this initial stage of relocalization, the abundance of the foregut sub-population is not as high as seen in the wild-type population shown in Fig 6A and thus, should not be expected to be as evenly distributed. What Fig 5D shows is similar to the relatively small sub-populations of *Vibrio* cells regaining swimming motility during the motility gain-of-function experiments shown in Fig 7A '24h', S9 Mov (starting at 00:05), and S10 Mov (starting at 00:18). These additional data show that *Vibrio* can form tight and focused groups of swimming cells near the anterior wall of the foregut. Therefore, our current interpretation of the data presented in Fig 5D is that within the first several hours of inducing Δche^{GOF}, which based on Fig 5E has not recovered wild-type levels of abundance, a moderately-sized sub-population emerges and is in the process of localizing to the foregut region and has yet to grow and fill this region as is the case for wild type.

7) *line 437 Nice control and follow up. This rapid evolution story is an interesting result hidden in this already substantial body of work.*

RESPONSE: We appreciate the positive feedback on these experiments. We are currently working to understand this process in more detail.

8) *Do the Δmot and Δche strains produce normal levels of flagellin on a per cell basis? Flagella appear normal in S1, but do all cells look that way? Could you do a Western? NOT necessary to do this for publication.*

<u>RESPONSE</u>: This is an important point and needs to be considered in future work, both in the context of understanding the in vivo aggregation behavior of motility mutants and *Vibrio*'s proinflammatory activity. It is difficult to estimate the per cell production of flagellin/flagella using our current SEM data. We intentionally prepared and imaged low-density cell suspensions to make sure we could capture single cells, which means only a low number of cells in total were imaged. In addition, it is conceivable that the process of preparing cells for imaging by SEM could have inadvertently sheared flagella from cells at some low rate. We did in fact see both flagellated and a subset of non-flagellated cells for each strain. To convey the possibility that flagellin expression and flagellar assembly could be playing a role in our experiments we made the following revision to the text within the discussion section:

(p22, lines 594–595)
"It is also possible that the Δmot and Δche mutants differ from wild-type in their level of expression of inflammatory flagellar components despite displaying intact flagella in vitro."

9) *In Fig 6A., Δche looks like it has anterior localization. Is this normal for Δche?*

<u>RESPONSE</u>: Yes, the pattern of Δche localization depicted in Fig 6A is typical and consistent with other data presented in our study. As described in our response to comment #6 above, in Fig 2C 'Δche' and in the text on p9, lines 244–245 we noted that Δche forms an adherent (or closely associated) layer of cells along the anterior wall of the foregut, which can also be seen in S1 Mov (starting at 00:18) and S2 Mov (starting at 00:38). This layer of cells can fluctuate in abundance, and in Fig 6A this population is slightly bigger than what we typically observe, but it is not out of the ordinary.

10) *It could be interesting to do scRNAseq on the activated TNFa-GFP+ liver cells to determine the cell type.*

<u>RESPONSE</u>: We agree. We have elaborated on this point further within the discussion:

(p 21, lines 576–584)
"On the host side, our work implicates macrophages as a host cell type that is capable of responding to *Vibrio*'s motile behavior through the upregulation of TNFα, but whether the mechanism of sensing *Vibrio* is direct or indirect remains to be determined. We also observed other host cells at systemic sites, particularly within the liver, that upregulate

TNFα in response to wild-type *Vibrio* colonization. Transcriptional profiling (e.g., via single-cell RNA sequencing) and transgenic animals carrying genetic reporters will be useful for probing the identity of these additional host cell types as well as the receptor(s) and signaling cascades involved in sensing *Vibrio* populations."

Reviewer 2 comments

1) *One of the strengths of this study is the careful and clever genetics, comparing mutants with very specific defects in chemotaxis and motility (that leave the flagellum intact). The authors might want to consider analyzing a mutant that is defective in producing flagella altogether. The aggregation phenotype observed in the mot and che mutants might be due to non-productive expression of flagella and a completely aflagellate mutant might not display the phenotypes observed with the mot and che mutants.*

RESPONSE: We agree, this is certainly a possibility that needs to be explored in future work. To help call attention to this point, we have added the following revised text within the discussion:

(p22, lines 603–605)
"It is also possible that the aggregation of Δmot and Δche stems from a defect in flagellar assembly or signaling, which could be disentangled using mutants lacking flagella altogether"

2) *Line 99-129: The last two paragraphs of the introduction are mostly a summary of the findings presented later in the paper; I think this section can be condensed significantly without loss of clarity to the reader.*

RESPONSE: In our revised manuscript, we have merged and condensed the final two paragraphs of the introduction.

(p5, lines 107–122)
"In the present work we used *Vibrio* as model gut symbiont to investigate the mechanisms by which its motility behaviors control its colonization and contribute to its proinflammatory potential. By combining live imaging, host and bacterial mutants, and in situ manipulation of motility behaviors, we were able to disentangle *Vibrio*'s requirements for motility during multiple stages of intestinal colonization. We found that for *Vibrio*, swimming motility and chemotaxis do not enhance exponential growth rate but rather enable cells to physically resist intestinal expulsion and maintain stable, highly abundant populations. We also found that host tissues—namely, gut-associated macrophages and cells within the liver—are acutely sensitive to bacterial motility and spatial organization within the intestine. Together,

our work expands the scope of bacterial swimming motility during intestinal colonization by revealing how motility can shape the large-scale spatial organization and dynamics of gut bacterial populations. Our study further shows that intestinal mechanics are a host spatial control measure capable of regulating the abundance and persistence of gut bacteria. Ultimately, our work yields new mechanistic insights into the form and function of the intestinal ecosystem and highlights that bacterial motility and the factors controlling the spatial organization of resident microbiota are potential targets for therapeutic manipulation of the gut microbiome."

3) *Consistent with standard nomenclature in the field, gene name/symbols should be italicized when denoting mutations.*

RESPONSE: We assume that the reviewer is referring to our use of "Δmot" and "Δche" to indicate our mutant strains. Here, our intent was to simplify the mutant names by referring to their affected *behavior* rather than their mutated *gene*. We find that using gene names can be cumbersome to read and hamper accessibility. In a similar way, others have used a trait-based naming approach to indicate, as in one example, bacterial mutants with a defective Type 6 secretion system (i.e., ΔT6SS) (Ho et al, *PNAS*, 2017; PMID: 28808000). We note that we conform to standard genetic nomenclature when referring to specific mutant loci and within the Methods section and S1 Table where our bacterial strains are described. We appreciate the reviewer's comment and have attempted to make the intent of our naming scheme clearer with the following revisions to the text where we first introduce our strains (underlined text indicates specific revisions):

(p6, lines 129–134)
"To test swimming motility in general, we deleted the two-gene operon *pomAB* that encodes the polar flagellar motor (creating a swimming motility-deficient *Vibrio* mutant we refer to as "Δmot"). To test *Vibrio*'s ability to spatially organize its populations in response to environmental cues, we deleted the gene *cheA2*, which encodes a histidine kinase necessary for chemotaxis (creating a chemotaxis-deficient *Vibrio* mutant we refer to as "Δche")."

4) *Generally, the presentation of the data is outstanding. The only thing I was confused about was what was measured in Fig. 1C (first thought was Vibrio), maybe labelling the y axis with "Aeromonas population" would help.*

RESPONSE: We can see how this graph may have been confusing. In fact, both *Aeromonas* and *Vibrio* abundances are plotted from the indicated competition schemes denoted on the x-axis. To clarify what is plotted in Fig 1C, we have added the revised text to the figure legend as follows:

(p7, lines 160–162)
"Intestinal abundances of *Aeromonas* and wild-type or mutant *Vibrio* strains during different competition schemes. *Aeromonas* abundances while alone during mono-association are shown for statistical comparison."

5) *The authors should consider citing previous work from the Salmonella field. This previous work, although on a pathogenic organism, is in line (and strengthens) the findings in the current paper. The Hardt lab had shown that Salmonella mutants deficient for chemotaxis are delayed for inducing inflammation (PMID: 15213159). The Baumler lab reported that energy taxis towards nitrate is important for invasion of Peyer's patches (PMID: 27435462).*

RESPONSE: We have considered the reviewer's advice and revised our introduction to call attention to the progress that has been made in understanding the role of motility in the context of infectious bacteria (revised text is also pasted below). We note that we have cited the following articles that are relevant to the *Salmonella* field:

Ref 17. Stecher/Hardt: *Motility allows S. Typhimurium to benefit from the mucosal defense* (PMID: 18241212)

Ref 18. Rivera-Chavez/Baumler: *Energy taxis toward host-derived nitrate supports a salmonella pathogenicity island 1-independent mechanism of invasion* (PMID: 27435462)

Ref 19. Stecher/Hardt: *Flagella and chemotaxis are required for efficient induction of Salmonella enterica serovar typhimurium colitis in streptomycin-pretreated mice* (PMID: 15213159)

Ref 22. Zeng/Neish: *Flagellin Is the Major Proinflammatory Determinant of Enteropathogenic Salmonella* (PMID: 14500664)

(p3, lines 63–75)
"The connection between motility and gastrointestinal colonization has historically been studied in the context of pathogens such as *Helicobacter pylori*, *Campylobacter jejuni, Vibrio cholerae*, and *Salmonella* Typhimurium [12]. With *S*. Typhimurium in particular, it has been found that this pathogen uses motility and chemotaxis to associate with and invade the intestinal mucosa, induce inflammation, and facilitate growth [17–19]. In several instances it has also been shown that flagellin, the protein subunit comprising the bacterial flagellum, can be a major driver of inflammation [20–22]. Highlighting the importance of curbing the pathogenic potential of motile bacteria, studies in mice have revealed several mechanisms by which hosts detect and quench flagellar motility to maintain intestinal homeostasis [4,20,23,24]. In total, significant progress has been made in understanding the

role motility plays in the infectious lifestyles of pathogens and its proinflammatory consequences for the host. However, a broader view of how motility behaviors might be shaping the lifestyles of resident gut bacteria remains limited and largely unexplored."

Reviewer #3 comments

1) *Fig 2C and 2F, S1 and S2 mov, page 8, lines 196-212: While it is clear in the movies that the Δmot bacterial strain is not motile, it is not so clear that there is a difference in the WT and Δche in the areas of high fluorescence. Fig 2C WT points to an area of "dense population of motile and planktonic cells" while Fig 2C Δche points to an area that looks very similar that is labeled "dense aggregate of non-motile cells". In the movies you only see motile or highly fluorescent regions. Therefore, how was the graph in Fig 2F determined? Materials and methods does not really make this clear how this was determined.*

RESPONSE: Thank you for calling attention to this potentially confusing point. First, in previous reports we have published movies showing that the dense masses of wild-type cells in the foregut are composed of planktonic and swimming cells. For example, Movie S1 in Wiles et al, 2018 (PMID: 30301859) and Video S1 in Wiles & Schlomann et al, 2018 (PMID: 30448038). Movie S1 can be freely viewed on Figshare: https://doi.org/10.6084/m9.figshare.7040309.v1. In Fig 2C in the current manuscript, we show a static image revealing the planktonic nature of wild-type *Vibrio* cells within these dense masses, which was captured by sparse labeling of the *Vibrio* population as noted in the original figure Fig 2 legend: "*Top right inset shows a zoomed-in view of wild-type Vibrio cells in a separate fish that was colonized with a 1:100 mixture of green- and red-tagged variants so that the cellular organization of the dense Vibrio population could be discerned. The dilute channel (green) is shown.*"

To better communicate the motile and planktonic nature of wild-type *Vibrio* we have revised the text and included a direct link to the published observations on Figshare (underlined text is revised):

(p9, lines 227-232)
"In line with our previous characterizations [26,27], wild-type *Vibrio* assembled dense populations concentrated within the foregut that were almost entirely composed of planktonic cells swimming in the lumen as well as within the intestinal folds (Fig 2C–2G, S1 and S2 Mov). A movie representation of the static image presented in Fig 2C "wt" under "foregut region" was previously published and can be viewed here: https://doi.org/10.6084/m9.figshare.7040309.v1 [26]."

To better highlight that the Δche mutant is aggregated within the midgut, we have appended a live movie of this strain within midgut region to S1 Mov. These new data show a large, rigid aggregate of Δche in the context of intestinal tissues undergoing low-amplitude contractions with a few motile Δche cells swimming outside the aggregate. We refer to these new data through the revised text within the results and S1 Mov caption as follows:

(results, p9, lines 238-239)
"In S1 Mov we provide a live representation of Δche within the midgut to emphasize its aggregated form within this intestinal region."

(caption, p47, lines 1392-1396)
"The first three fields of view shown center on the foregut region. The fourth field of view ("che-midgut") centers on the midgut to highlight aggregates of Δche cells within this region. For the fourth field of view, intensities were log-transformed to highlight both the structure of the aggregates and the motile, planktonic cells visible in the lower left."

To address the reviewer's question about how data plotted in Fig 2F were determined, we have added the following section within the Methods:

(methods, p34, lines 959-969)
"Measurement of planktonic fractions

Planktonic fractions were computed for each fish by dividing the number of identified single cells by the total abundance. For wild-type *Vibrio*, the primary population of motile bacteria is typically too dense for us to resolve single cells. However, sparse labelling experiments, in which fish were colonized with GFP- and dTomato-marked *Vibrio* at a ratio of 1:100 (Fig. 2C, top row, right), indicated that this population was indeed entirely planktonic. Movies showing motility of sparsely labelled cells were previously published [26,27]. Therefore, to compute planktonic fraction for wild-type *Vibrio* in single-labeled populations, we treated the dense, anterior sub-population computationally just like we would an aggregate—using our aggregate detection algorithm and estimating the number of cells present by normalizing the total fluorescence intensity to the mean single cell intensity—but then counted these cells as planktonic."

2) *Page 14, lines 390-395: What is the delineation between "foregut region" and "intestinal tissues"? The authors state that 54% of the tnfa-positive cells in the foregut were macrophages, but 93% of tnfa-positive cells that were associated with intestinal tissues were macrophages. Isn't the foregut considered part of the intestinal tissue? Please clarify what is meant here.*

RESPONSE: Thank you for calling attention to this point of confusion. Our intent was to define the "foregut region" as the field of view captured in our images that contains the foregut and adjacent tissues (which includes the liver). We note that on p17, line 468 in the original manuscript we wrote: "*Quantifying fluorescence intensity across the foregut region (including extraintestinal tissues and the liver)*…" In addition to this original text, we have attempted to further clarify our intent within the section describing the quantification of *tnfa*-positive macrophages by inserting the following revised text (underlined text has been revised):

(p18, lines 499–506)
"Using double transgenic zebrafish carrying the *tnfa* reporter and expressing fluorescently marked macrophages (Tg(*mpeg1*:mCherry) [46]), we quantified the fraction of *tnfa*-positive macrophages within the foregut region, which we consider here as a field of view containing the foregut and adjacent tissues such as the liver. We found that ~half (54 ± 10% [mean ± standard deviation, n = 100 cells from 4 animals]) of the *tnfa*-positive cells in the foregut region induced by *Vibrio* were indeed macrophages (Fig 6D and S9 Mov). Nearly all *tnfa*-positive cells that were directly associated with the foregut were macrophages (93 ± 12% [mean ± standard deviation, n = 18 cells from 3 animals])."

3) *Page 14, lines 395-398, Figure 6D: The authors state that in contrast to tnfa-positive macrophages in the foregut, the tnfa-positive cells found in the liver area were not macrophages or neutrophils. However, the "tnfa-positive macrophage" that the arrowhead is pointing to the in Fig 6D, looks just like 3 other cells that should be "tnfa-positive macrophage" outside the foregut region, presumably associated with the liver. Can you clarify this discrepancy?*

RESPONSE: We appreciate the reviewer's comment. Our intent was not to convey that *none* of the cells found associated with the liver could be macrophages. In fact, due to the migratory nature of macrophages, it is likely that *tnfa*-positive macrophages would be found traversing liver tissues, as might be the case for the two anterior-localized cells in Fig 6D that we think the reviewer is specifically referring to. We note that we are currently working to understand the dynamic responses of cells and tissue compartments to *Vibrio* in more detail.

To help clarify this point, on p18, line 506 we now write (underlined text is revised): "In contrast, the majority of *tnfa*-positive cells associated within the liver did not appear to be macrophages…"

4) *Discussion: One issue that did not seem to be addressed anywhere in the paper, but probably should have been in the discussion was how the lack of chemotactic ability would cause a similar defect in persistence as the motility mutant. The authors show that the Δche mutant was still mobile in the intestine, so it is not just motility that is causing the defect. How would the chemotaxis of the WT strain in the gut allow it to persist? What is the WT strain sensing and/or how is chemotaxis keeping the WT from aggregating? The authors should discuss possibilities as to why this would be the case.*

RESPONSE: We agree that we need to elaborate on this important point. As we wrote in our responses to similar comments made by Reviewers 1 and 2, we have now included the following revised text within the discussion:

(p22, lines 600–618)
"The aggregation of Δmot and Δche within the intestine was unexpected, especially for Δche, which displays vigorous swimming in vitro. It is possible that both mutants experience shifts in metabolism or induce biofilm behaviors in vivo as a consequence of their inability to effectively control their spatial distribution. It is also possible that the aggregation of Δmot and Δche stems from a defect in flagellar assembly or signaling, which could be disentangled using mutants lacking flagella altogether. Alternatively, Δmot and Δche cells may become entrapped within intestinal mucus and grow locally to produce small clonal aggregates that are subsequently consolidated into larger aggregates by intestinal mechanics before being collectively expelled. Corroborating this idea, it was shown in an infant mouse model that the attenuated colonization of a human-derived isolate of *V. cholerae* lacking motility can largely be reversed in animals that are pre-treated with a mucolytic agent that disrupts mucus architecture [56]. Furthermore, it is possible that the swimming activity of Δche may actually facilitate mucus entrapment. It has been shown that non-chemotactic, straight-swimming bacterial cells that are unable to periodically redirect their swimming trajectory can become jammed within a porous medium (such as in a soft agar matrix commonly used to study chemotaxis in vitro) [57]. We propose that a similar mechanism could lead to entrapment and aggregation of Δche cells within intestinal mucus, which wild-type *Vibrio* cells avoid because they are able to actively escape from mucus through regular changes in swimming direction mediated by chemotactic signaling."

Reviewer #4 comments

1) *The movies in WT zebrafish showing expulsion of the delta-mot and of the delta-che mutants are impressive. Are movies available from the ret-/- mutant animals, showing that the detal-mot and delta-che mutant are rescued? Also, Fig S2B only shows the delta-mot mutant, but not the delta-che mutant, in ret-/- mice. What is the phenotype of the delta-che mutant in these animals?*

<u>RESPONSE</u>: We did not capture movies of motility or chemotaxis mutants in *ret* mutant animals, but we can comment that visual inspection of the overall biogeography did not show anything unexpected (i.e., we observed cells throughout the *ret* intestine in most cases and for Δmot we could observed cells within the anterior foregut). We also note that we are actively investigating colonization dynamics in *ret* mutant animals. As for the phenotype of Δche in *ret* mutant animals, we did not capture 3D scans because the pattern of localization for Δche is largely the same except that there are simply more cells within the intestine. The intent of S2B Fig was to point out that for Δmot there is a clear localization change that can be observed in some *ret* mutant animals that is not detected in wild-type animals. Because Δche occupies numerous regions of the intestine already, changes in localization would not be as clear.

2) *Ln 66: "much remains unknown about how motility and behaviors promote intestinal colonization and provide bacteria a competitive advantage." In the context of host-pathogen interaction, work with Salmonella has shown the importance of motility and chemotaxis to promote intestinal colonization, to access nutrient sources in the mucus layer, and to outcompete the gut microbiota. I think it would be important to discuss the work of Stecher and Hardt, and Rivera-Chavez and Baumler, in the context of this new study.*

<u>RESPONSE</u>: We agree. We have revised our introduction (provided below) to highlight the progress that has been made in understanding the roles of motility and chemotaxis during intestinal colonization in the context of infectious bacterial pathogens.

(p3, lines 63–75)

"The connection between motility and gastrointestinal colonization has historically been studied in the context of pathogens such as *Helicobacter pylori*, *Campylobacter jejuni, Vibrio cholerae*, and *Salmonella* Typhimurium [12]. With *S*. Typhimurium in particular, it has been found that this pathogen uses motility and chemotaxis to associate with and invade the intestinal mucosa, induce inflammation, and facilitate growth [17–19]. In several instances it has also been shown that flagellin, the protein subunit comprising the bacterial flagellum, can be a major driver of inflammation [20–22]. Highlighting the importance of curbing the pathogenic potential of motile bacteria, studies in mice have revealed several mechanisms by which hosts detect and quench flagellar motility to maintain intestinal homeostasis [4,20,23,24]. In total, significant progress has been made in understanding the role motility plays in the infectious lifestyles of pathogens and its proinflammatory consequences for the host. However, a broader view of how motility behaviors might be shaping the lifestyles of resident gut bacteria remains limited and largely unexplored."
